# The Genetics of Primary Ciliary Dyskinesia in Puerto Rico

**DOI:** 10.3390/diagnostics12051127

**Published:** 2022-05-02

**Authors:** Wilfredo De Jesús-Rojas, José Muñiz-Hernández, Francisco Alvarado-Huerta, Jesús M. Meléndez-Montañez, Arnaldo J. Santos-López, Ricardo A. Mosquera

**Affiliations:** 1 Department of Pediatrics–Anatomy and Neuroanatomy, University of Puerto Rico, Medical Sciences Campus, San Juan, PR 00921, USA; alvarado-huerta.f@northeastern.edu; 2 Department of Pediatrics, Ponce Health Science University, Ponce, PR 00716, USA; jmelendez20@stu.psm.edu (J.M.M.-M.); asantos18@stu.psm.edu (A.J.S.-L.); 3 Department of Natural Science, University of Puerto Rico, Cayey Campus, Cayey, PR 00736, USA; jose.muniz8@upr.edu; 4 Department of Pediatrics, McGovern Medical School, University of Texas Health Science Center at Houston, Houston, TX 77030, USA; ricardo.a.mosquera@uth.tmc.edu

**Keywords:** primary ciliary dyskinesia, *RSPH4A*, pathogenic, variant of uncertain significance, Puerto Rico, founder mutation

## Abstract

Primary ciliary dyskinesia (PCD) has been linked to more than 50 genes that cause a spectrum of clinical symptoms, including newborn respiratory distress, sinopulmonary infections, and laterality abnormalities. Although the *RSPH4A* (c.921+3_6delAAGT) pathogenic variant has been related to Hispanic groups with Puerto Rican ancestry, it is uncertain how frequently other PCD-implicated genes are present on the island. A retrospective chart review of *n* = 127 genetic reports from Puerto Rican subjects who underwent genetic screening for PCD variants was conducted from 2018 to 2022. Of 127 subjects, 29.1% subjects presented PCD pathogenic variants, and 13.4% were homozygous for the *RSPH4A* (c.921+3_6delAAGT) founder mutation. The most common pathogenic variants were in *RSPH4A* and *ZMYND10* genes. A description of the frequency and geographic distribution of implicated PCD pathogenic variants in Puerto Rico is presented. Our findings reconfirm that the presence of PCD in Puerto Rico is predominantly due to a founder pathogenic variant in the *RSPH4A* (c.921+3_6delAAGT) splice site. Understanding the frequency of PCD genetic variants in Puerto Rico is essential to map a future genotype-phenotype PCD spectrum in Puerto Rican Hispanics with a heterogeneous ancestry.

## 1. Introduction

Primary ciliary dyskinesia (PCD) is a rare genetic disease that causes a spectrum of oto-sinopulmonary symptoms secondary to more than 50 implicated genes that result in ciliary dysmotility [1,2]. PCD can lead to chronic bacterial colonization of the upper and lower airways, recurrent otitis media, and in some cases, infertility and laterality defects [3]. At least two of four cardinal features, including organ laterality defects; unexplained neonatal respiratory distress; year-round, daily, wet cough; and year-round nasal congestion, increase the likelihood of PCD diagnosis as per the American Thoracic Society (ATS) clinical guidelines [4,5]. Recently, the global prevalence of PCD has been estimated to be one in 7554 individuals, and in Hispanics, the prevalence is one in 16,309 individuals [6]. Genetic testing plays an essential role in evaluating PCD in countries where nasal nitric oxide (nNO) and transmission electron microscopy (TEM) are limited or not available [7]. A founder mutation in the *RSPH4A* (c.921+3_6delAAGT) was confirmed in native individuals living in the United States and Puerto Rico [7,8].

Due to genetic drift and the subsequent population expansion, the island of Puerto Rico has a heterogeneous genetic composition [9]. The topography of an island that is 100 miles long by 35 miles wide and surrounded by a central mountain range promotes the isolation and risk of consanguinity of communities in specific regions in Puerto Rico. In the past, other autosomal recessive disorders have been more frequent in the northwest region of the island [10]. Today, the geographic distribution and frequencies of PCD related variants are still unknown. A previous study suggested that the *RSPH4A* (c.921+3_6delAAGT) founder mutation was brought by European conquistadors and distributed across the island [11]. Similarly, other founder pathogenic mutations may be present in our population. The frequency of variants of unknown significance (VUS) and their clinical role in PCD diagnosis remain debatable [12]. Identifying VUS frequency in ciliary-related genes across different populations may help understand their role in PCD as a spectrum disorder [13]. Although the prevalence of PCD in Puerto Rico is still unknown, an analysis of the geographic dissemination of pathogenic variants across different healthcare regions could provide a better idea of genetic distribution for PCD across the 78 municipalities. Our study presents the geographic frequency and distribution of PCD implicated genetic variants in Puerto Rico in a cohort of subjects screened for PCD. In addition, the demographics and clinical characteristics for 17 homozygous patients with the *RSPH4A* (c.921+3_6delAAGT) founder mutation are presented.

## 2. Materials and Methods

**Figure 1:**
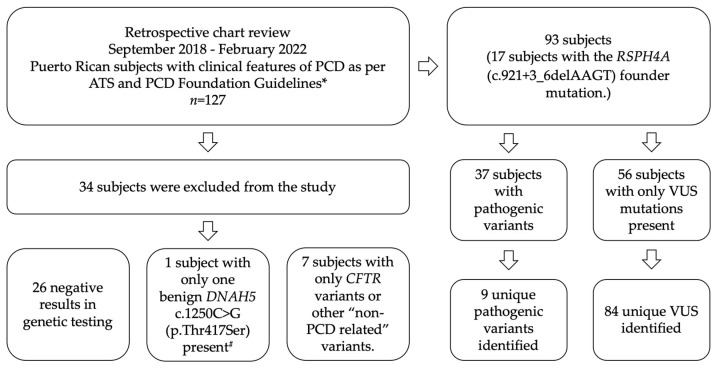
Diagram of inclusion and exclusion criteria. * Subjects with pathogenic variants or VUS in PCD genes were included in the study. ^#^ *DNAH5* c.1250C > G (p.Thr417Ser) benign variant was excluded.

A descriptive retrospective chart review of *n* = 127 Puerto Rican subjects previously screened for PCD by genetic testing was completed. Fifteen subjects were siblings from four different families. The retrospective study was conducted between September 2018 and February 2022 in an outpatient community clinic with expertise in rare pulmonary disorders, recently accredited as a PCD Center by the PCD Foundation (Figure 1). Analysis of genetic reports was completed in subjects that previously were evaluated by a physician following the diagnostic algorithm suggested for the PCD diagnosis, and it was part of the differential diagnosis [4]. Subjects with pathogenic variants or VUS in PCD genes were included in the study. Subjects with genetic variants related to other ciliopathies or cystic fibrosis were excluded. Saliva or buccal swabs samples were analyzed by a commercial genetic testing laboratory (Invitae Corporation, San Francisco, CA, USA). Complete genetic sequence analysis for 42 PCD-related genes, including deletions and duplications associated with the *CFTR* gene, was conducted (Table A1). Only pathogenic variants or VUS were considered in our cohort. All PCD genetic variants were considered and included in the study analysis. VUS variants were defined as genetic sequences for which there is an unclear association to disease today due to the lack of information. A pathogenic variant was defined as an illness-causing disease previously reported on PCD in the population. In-silico computational analysis as presented by Invitae’s Sherloc variant interpretation framework was included. Sherloc variant interpretation framework relies on a point-based evidence scoring system built upon the joint consensus guidelines from the American College of Medical Genetics and Genomics and the Association for Molecular Pathology [14].

Descriptive mapping of the PCD frequency of pathogenic variants was compiled and segregated across the seven healthcare regions per the Puerto Rico department of health (Metro, Caguas, Bayamón, Arecibo, Fajardo, Ponce, and Mayagüez). Metro and Bayamón regions have more access to specialized healthcare services due to the proximity to the capital of San Juan to urban areas. Arecibo, Fajardo, Ponce, and Mayaguez regions are mainly composed of municipalities with mainly rural areas with limited access to specialized health care. The genetic variants identified were systematically organized according to the geographic region of the subject’s home address using zip codes for municipality allocation from data obtained from genetic reports. The data collection and analysis for the protection of human subjects was approved by the institutional review board (IRB) from the University of Puerto Rico, Medical Sciences Campus in San Juan, Puerto Rico (B1730120).

## 3. Results

Of 127 subjects, 37 (29.1%) subjects presented PCD pathogenic variants, and 17 (13.4%) subjects were homozygous for the *RSPH4A* (c.921+3_6delAAGT) founder mutation. Thirty-four subjects were excluded due to negative genetic test results or cases where genetic variants were reported for other non-PCD ciliopathies or cystic fibrosis. A benign variant on *DNAH5* c.1250C > G (p.Thr417Ser) was excluded.

### 3.1. Distribution of PCD Genetic Variants in Puerto Rico

**Figure 2:**
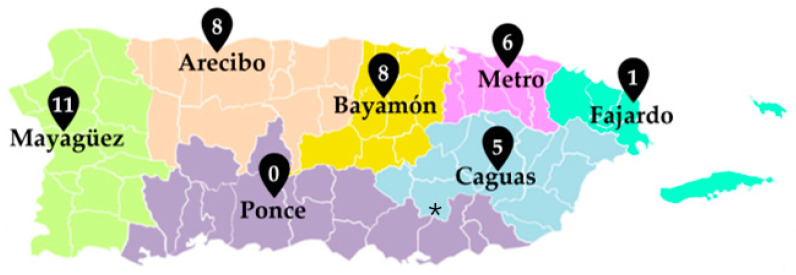
Geographic distribution of PCD pathogenic variants in Puerto Rico. Of the seven designated health regions in Puerto Rico, Mayagüez showed the highest frequency in pathogenic variants with 11 subjects. Two cases presented two different pathogenic variants in the same subject. * PCD clinic location.

Out of the 37 cases of pathogenic genetic variants associated with PCD within the seven designated health regions in Puerto Rico, Mayagüez was most prevalent with eleven (29.7%) cases. The closest regions were Bayamón (21.6%) and Arecibo (21.6%) with eight subjects, followed by Metro with six (16.2%), Caguas (13.5%) with five, and Fajardo (2.7%) with one case. Finally, the Ponce region showed no PCD pathogenic variant (Figure 2).

**Figure 3:**
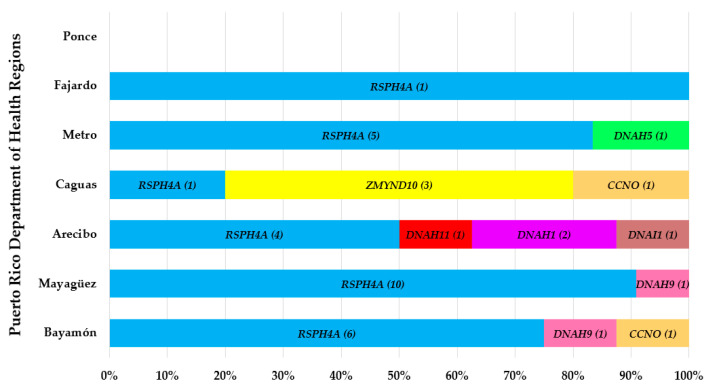
Percentage and number of total allele frequency of PCD pathogenic variants in Puerto Rico. The pathogenic variant (c.921+3_6delAAGT) in the *RSPH4A* gene was the most frequent.

The genetic distribution showed a considerably increased frequency of the *RSPH4A* (c.921+3_6delAAGT) pathogenic variant. Alleles for the *RSPH4A* (c.921+3_6delAAGT) pathogenic variant were found 27 times throughout the seven healthcare regions in Puerto Rico (Figure 3). Mayagüez was the region with the highest frequency for the *RSPH4A* (c.921+3_6delAAGT) pathogenic variant showing above 90% of the total pathogenic alleles found. The frequency of the total *RSPH4A* pathogenic alleles in other regions was found as follows: Bayamón (75%), Arecibo (50%), Metro (83%), and Fajardo (100%) regions. The Ponce region had 0% frequency for the *RSPH4A* (c.921+3_6delAAGT) pathogenic variant or any other variant associated with PCD.

**Figure 4:**
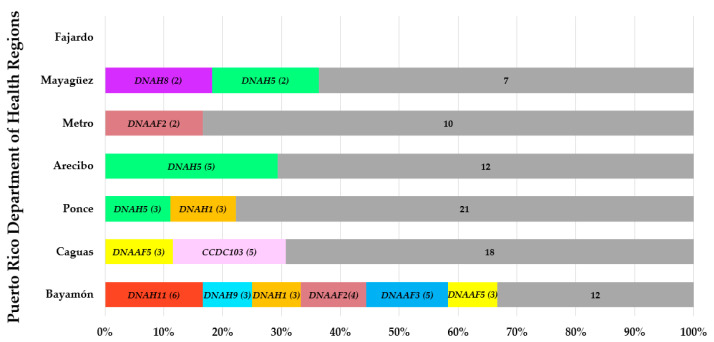
Percentage and numbers of total allele frequency of VUS in PCD genes in Puerto Rico. Gray bars show genetic variants with a frequency of less than 1.5% reported across regions.

In the Caguas region, the *ZMYND10* (c.85T > C (p.Ser29Pro)) pathogenic variant was found to be most frequent at 60%. Two other pathogenic variants had a slightly increased frequency; this includes the *RSPH4A* (c.921+3_6delAAGT) by 20% and *CCNO* (c.875_897del (p.Asp292Alafs*71)) by 20%. Arecibo region showed a 25% frequency of the *DNAH1* (c.10468_10471del (p.Arg3490Glnfs*4)) pathogenic variant. The only *DNAH5* pathogenic mutation was found in the Metro region. The *CCNO* (c.875_897del (p.Asp292Alafs*71)) pathogenic variant was found between Caguas and Bayamón. There were two pathogenic variants in the *DNAH9* gene found in Mayagüez (0.09%) and Bayamón (0.125) regions. The remaining two identified genes, *DNAH11* and *DNAI1*, were found less frequent in the Arecibo region. VUS in the PCD genes *DNAH5*, *DNAH11*, *DNAAF5*, *DNAAF2*, *DNAAF3*, *CCDC103*, *DNAH9*, *DNAH1*, and *DNAH8* were the most identified (Figure 4).

### 3.2. Frequency Percentage by Zygosity

To better understand the distribution of PCD-related variants, we considered analyzing the total frequency of individual genetic variants by looking at their frequency, including homozygous, heterozygous, and compound heterozygous.

#### 3.2.1. Pathogenic Frequency in PCD Implicated Genes

Regarding the frequency of PCD implicated pathogenic variants, the *RSPH4A* (c.921+3_6delAAGT) variant was the highest in our cohort, exhibiting a 66.7% frequency. If the *RSPH4A* (c.1103T > G p.Val368Gly) gene variant is taken into consideration (2.6%), the *RSPH4A* gene represents 69.3% of all pathogenic variants in Puerto Rico implicated in PCD. *ZMYND10* (c.85T > C (p.Ser29Pro)) pathogenic variant was the second most frequent, with 7.7%. *CCNO* (c.875_897del (p.Asp292Alafs*71)), *DNAH1* (c.10468_10471del (p.Arg3490Glnfs*4)), and *DNAH9* (c.308dup (p.Leu104Profs*45)) pathogenic variants all exhibiting a 5.1% frequency in our cohort. Mutations in *DNAH11*, *DNAH5*, and *DNAI1* heterozygous pathogenic variants represented 2.6% frequency, respectively. As a reference, the allele frequency of PCD pathogenic variants found in Puerto Rico compared to Latinos, and the general population is presented in Table 1. As a reference, the allele frequency of the most common PCD VUS found in Puerto Rico compared to Latino, and the general population is presented in Table 2. A complete list of all VUS is presented in Table A2.

#### 3.2.2. Homozygous Subjects with the *RSPH4A* (c.921+3_6delAAGT) Founder Mutation

Seventeen subjects were homozygous for the *RSPH4A* (c.921+3_6delAAGT) founder mutation in our cohort. Bronchiectasis was present in 15 (88%) subjects and was more frequent in adults (100%) than in children (71%). No laterality defects were identified in our cohort (0%). Forced expiratory volume in 1 s (FEV1) was lower in adults (44 + 8.8) as compared with children (67 + 25.2). Year-round wet cough and daily nasal congestion were found in all subjects (100%). The clinical characteristics and demographics of homozygous subjects for the *RSPH4A* (c.921+3_6delAAGT) founder mutation are summarized in Table 3.

## 4. Discussion

In Hispanics, the genetic information available regarding PCD is limited. The frequency of the PCD genes and the associated genetic variants in Puerto Rico were largely unknown. We studied a large cohort of 127 subjects who completed genetic screening for PCD as part of the differential diagnosis considering patient history per the ATS clinical guidelines [5]. Genetic characterization and the geographic distribution of subjects with suspected PCD were retrospectively analyzed to comprehend the frequency and extent of PCD across Puerto Rico. Descriptive clinical and demographic data for 17 subjects with the *RSPH4A* (c.921+3_6delAAGT)founder mutation is presented.

Analysis of the distribution of PCD pathogenic variants in our cohort in Puerto Rico found a genetic frequency of the *RSPH4A* (c.921+3_6delAAGT)across almost all healthcare regions in Puerto Rico. Interestingly, there were 11 subjects with pathogenic variants in the Mayagüez region, a well-known region with an increased frequency of autosomal recessive disorders due to the consanguinity of families. Out of fifteen in this region, only five municipalities proved to have subjects with a pathogenic variant for PCD. The same pattern was identified in the Bayamón region, where, out of eleven municipalities that compose this region, five have subjects with PCD pathogenic variants. We could argue about the presence of areas of increased incidence or hotspots for PCD in the Mayagüez and Bayamón regions, but additional research is needed to confirm our observations. No pathogenic variants were identified in the Ponce region. A hypothesis for his observation may be related to the fact that since the last years, the accessibility to medical services for referral in this region is limited after the local impact of earthquakes, the pandemic, and hurricanes, which reduced the actual population in the region. A larger cohort may be required to address this observation.

Above 50% of PCD genes affected in Europe and North America are due to genetic mutation in *DNAH5*, *DNAH11*, *CCDC39*, and *CCDC40* genes [16]. Previous studies showed that in Hispanics, the most common pathogenic or likely pathogenic variants were associated with *DNAAF4*, *DNAH11*, *DNAH5*, *DNAAF3*, and *ODAD1* [6]. Comparing the identified PCD pathogenic variants within our cohort with those published by Hannah et al. shows that the PCD Puerto Rican genetic pool may not be similar to what was previously reported for Latinos [6]. Apart from the *RSPH4A* gene, the pathogenic variants reported were in the following PCD genes: *ZMYND10*, *CCNO*, *DNAH1*, *DNAH9*, *DNAI1*, *DNAH11*, and *DNAH5*. The *CCDC40* and *CCDC39* genes are linked with a clinical severe PCD phenotype in previous publications [17]. In our cohort, only 1.5% of the pathogenic variants were related to *CCDCD40*, which is positive considering the increased severe spectrum of pulmonary involvement [17]. In comparison with Europe and North America, pathogenic variants in two of the most common genes (*DNAH5* and *DNAH11*) were present in our cohort. Findings may suggest the importance of PCD genetic testing in specific geographic locations apart from the general ethnicity of the subject. Additional studies to explore and compare the geographic frequency of PCD pathogenic variants among different islands in the Caribbean should be conducted. Compared with the allelic frequency published in the gnomAD database, our frequencies were higher as expected. Due to the presence of the *RSPH4A* (c.921+3_6delAAGT) founder mutation in Puerto Rico, a higher frequency (0.667) was expected as compared with the general (0.00003204) and Latino allele frequency (0.0001738). Compared with gnomAD, higher than expected frequencies were identified on other PCD genes. A reason for this observation is that we reviewed the genetic results of a cohort screened for PCD as part of the differential diagnosis, which falsely elevated the frequency of PCD variants in our sample. A large study screening the general population for the *RSPH4A* (c.921+3_6delAAGT) pathogenic variant is needed to know the actual prevalence of this founder mutation in Puerto Rico.

Our Puerto Rican heritage is an admixture of European, African, and Taino natives [18]. A previous publication identified the presence of the *RSPH4A* (c.921+3_6delAAGT) founder mutation in Puerto Rico [7]. Excluding VUS, the reported carrier frequency of *RSPH4A* mutations was 1 in 632 individuals. By extrapolation of the carrier frequency to our Puerto Rican 2021 population of 2.8 million people living on the island, we can anticipate 4430 individuals who are carriers of *RSPH4A* mutations. Our data support the elevated frequency of the *RSPH4A* (c.921+3_6delAAGT) pathogenic variant in Puerto Rico. Additional *RSPH4A* genetic variants were also present. We found one subject with a heterozygous *RSPH4A* (c.1103T > G (p.Val368Gly)) pathogenic variant presenting as compound heterozygous subjects concomitant with the *RSPH4A* (c.921+3_6delAAGT) pathogenic variant. Interrelationships among different *RSPH4A* heterozygous variants are present in Puerto Rican subjects, resulting in a spectrum of PCD in Puerto Rico. This confirms the previous trend stating that the mutations in the *RSPH4A* gene are largely dominant within our cohort and PCD community on the island.

The presence of VUS for PCD genes in Puerto Rico was analyzed. Previous studies identified a more significant number of PCD genes with VUS [6]. The authors discussed the importance of more extensive databases to explore the role of VUS in different ethnicities and geographic locations. Puerto Rico is not an exception. Due to a heterogeneous genetic pool, 59.8% of the subjects in our cohort presented VUS. Analysis of additional subjects is needed to understand genetic variants in genes such as *DNAH8* that are linked to infertility, and current data are not clear about their role in the respiratory motile cilia [19].

Our study has limitations. The study retrospectively analyzed 127 individuals screened for PCD from one single PCD clinic. Additional pulmonary clinics in Puerto Rico with PCD patients were not included, and data from multiple clinics may expand or alter the genetic results of this study, even though our clinic serves as the only PCD referral center from other pulmonary clinics on the island for both adults and pediatrics with the capability to perform nNO using a chemiluminescence analyzer. Furthermore, genetic variants in more than 50 genes are linked with PCD, and we analyzed only 42 genes, including the *CFTR* gene. Genotype–phenotype studies are needed to understand the role of pathogenic variants and implicated VUS in the clinical setting or PCD diagnosis. Reanalysis of new data in the future may help to unmask new PCD genetic variants and classify VUS as disease-causing for the Puerto Rican population. Finally, existing classifications of VUS identified in this study may be reclassified as likely benign or likely pathogenic as more cases are identified in the near future.

## 5. Conclusions

Our study provides an overview of the actual geographic distribution of PCD pathogenic variants in Puerto Rico as well the clinical characteristics of the homozygous subjects with the *RSPH4A* (c.921+3_6delAAGT) founder mutation. Although the information presented may change in the future due to the increasing availability of genetic testing, we reconfirm the presence of the *RSPH4A* (c.921+3_6delAAGT) founder mutation as the primary variant responsible for PCD cases across the island. Our work provides a better understanding of the frequency and distribution of PCD implicated genes and genetic variants across the municipalities in Puerto Rico. Overall, the implicated PCD genes are specific for certain ethnicities such as Hispanics but also may vary among the same ethnic background as Puerto Ricans.

## Figures and Tables

**Table 1:** Frequency of PCD genes with pathogenic variants found in Puerto Rico.

		Allelic Frequency			In Silico Evidence ^#^	
PCD Genes	Pathogenic Variants	Puerto Rico	General *	Latino *	SIFT	PolyPhen-2
*RSPH4A*	c.921+3_6delAAGT (Intronic)	0.667	0.00003204	0.0001738	^^^ Aberrant splicing; absent or disrupted protein product.	
*ZMYND10*	c.85T > C (p.Ser29Pro)	0.077	0.00002682	0.0000586	Deleterious	Probably damaging
*CCNO*	c.875_897del (p.Asp292Alafs*71)	0.051	0.000003991	0.00	^^^ C-terminus disruption of the *CCNO* Protein. Likely to be disease-causative.	
*DNAH1*	c.10468_10471del (p.Arg3490Glnfs*4)	0.051	0.0000856	0.000198	^^^ Created a premature stop signal. Results in absence of disruptive protein.	
*DNAH9*	c.308dup (p.Leu104Profs*45)	0.051	0.0002849	0.0001783	^^^ Created a premature stop signal. Results in absence of disruptive protein.	
*RSPH4A*	c.1103T > G (p.Val368Gly)	0.026	0.000003978	0.00	Deleterious	Probably damaging
*DNAH11*	c.3133C > T (p.Arg1045*)	0.026	0.00001209	0.00	^^^ Created a premature stop signal. Results in absence of disruptive protein.	
*DNAH5*	c.2431+5G > A (Intronic)	0.026	0.00009564	0.00	^^^ Aberrant splicing	
*DNAI1*	c.370C > T (p.Arg124Cys)	0.026	0.000403	0.0003386	Tolerated	Probably damaging

* General and Latino allele frequency obtained from gnomAD database [15]. Available online: https://gnomad.broadinstitute.org/ (accessed on 21 March 2022). ^#^ In silico evidence reported by Invitae Corporation, San Francisco, CA, USA. SIFT, Sorting Intolerant From Tolerant; PolyPhen-2, Polymorphism Phenotyping v2. ^^^ Not Reportable.

**Table 2:** Frequency of PCD genes with VUS in Puerto Rico.

		Allelic Frequency			In Silico Evidence ***	
PCD Genes	Variants of Unknown Significance (VUS) *	Puerto Rico	General ^#^	Latino ^#^	SIFT	PolyPhen-2
*DNAAF2*	c.58G > C (p.Val20Leu)	0.053	0.00004059	0.0002017	Tolerated	Probably damaging
*DNAAF4*	c.186C > G (p.Ser62Arg)	0.045	0.00001193	0.00002895	Deleterious	Probably damaging
*DNAH11*	c.8888C > A (p.Ser2963Tyr)	0.038	**	**	Deleterious	Probably damaging
*DNAAF5*	c.969C > A (p.Asp323Glu)	0.038	0.00004926	0.0003222	Likely tolerated	Likely tolerated
*DNAAF3*	c.1672G > T (p.Glu558*)	0.038	0.00002413	0.00002899	^^^ Created a premature stop signal. Results in absence of disruptive protein.	
*SPAG1*	c.2717A > G (p.Asp906Gly)	0.023	0.00005179	0.0001737	Deleterious	Benign
*CCDC103*	c.82C > T (p.Arg28Trp)	0.023	0.00005142	0.0000315	Likely disruptive	Likely disruptive
*CCDC40*	c.1109G > A (p.Arg370His)	0.015	0.00009654	0.00006263	Deleterious	Probably damaging
*DNAI2*	c.1153G > A (p.Asp385Asn)	0.015	0.00003889	0.0001693	Deleterious	Probably damaging
*CCNO*	c.685G > A (p.Gly229Ser)	0.015	0.0002135	0.00007137	Deleterious	Benign
*RSPH4A*	c.902A > C (p.Gln301Pro)	0.015	0.00001598	0.00008691	Tolerated	Benign
*CFAP298*	c.803C > T (p.Pro268Leu)	0.015	0.00001768	0.0001129	Likely tolerated	Likely tolerated
*RSPH1*	c.824G > A (p.Arg275Gln)	0.015	0.00002831	0.00	Tolerated	Benign
*RSPH1*	c.673C > T (p.Pro225Ser)	0.015	0.0000283	0.0001411	Tolerated	Benign
*CCDC103*	c.146G > C (p.Gly49Ala)	0.015	0.00002829	0.00002822	Tolerated	Benign
*DNAH5*	c.6409C > A (p.Leu2137Ile)	0.015	0.00003537	0.0002259	Deleterious	Probably damaging
*DNAH5*	c.5413C > T (p.Arg1805Cys)	0.015	0.0001203	0.0001129	Deleterious	Probably damaging
*DNAH5*	c.2577+5T > G (Intronic)	0.015	0.00001993	0.00005791	Not reportable: aberrant splicing	
*DNAH5*	c.477C > T (Silent)	0.015	**	**	^^^ Create or strengthen a splice site.	
*DNAH5*	c.8398G > A (p.Val2800Ile)	0.015	0.00002786	0.00005793	Tolerated	Benign
*DNAAF5*	c.1471-3C > T (Intronic)	0.015	0.00000442	0.0000308	^^^ Aberrant splicing; absent or disrupted protein product.	
Other Genes		0.525				

* Zygosity of all VUS presented was heterozygous. ** Frequency not reported at gnomAD database. *** In silico evidence was reported by Invitae Corporation, San Francisco, CA, USA. SIFT, Sorting Intolerant From Tolerant; PolyPhen-2, Polymorphism Phenotyping v2. ^#^ General and Latino allele frequency obtained from gnomAD database [15]. Available online: https://gnomad.broadinstitute.org/ (accessed on 21 March 2022). ^^^ Not reportable.

**Table 3:** Clinical characteristics and demographics of homozygous subjects for the *RSPH4A* (c.921+3_6delAAGT) founder mutation.

Characteristics	Children (*n* = 7)	Adults (*n* = 10)	Total (*n* = 17)
Gender (female), *n* (%)	3 (42)	8 (80)	11 (64)
Age, median+SD, (years)	11+11.5	38+13.3	23+17.4
Ethnicity, *n* (%), Hispanic, Puerto Rican	7 (100)	10 (100)	17 (100)
Year-round wet cough, *n* (%)	7 (100)	10 (100)	17 (100)
Year-round, daily nasal congestion, *n* (%)	7 (100)	10 (100)	17 (100)
History of neonatal respiratory distress, *n* (%)	4 (57)	4 (40)	8 (47)
Hearing loss, *n* (%)	6 (85)	6 (60)	12 (71)
Bronchiectasis on HRCT, *n* (%)	5 (71)	10 (100)	15 (88)
Chronic secretory otitis media, *n* (%)	2 (29)	5 (100)	7 (41)
Laterality defects, *n* (%)	0 (0)	0 (0)	0 (0)
Forced expiratory volume in 1 s (FEV1), median + SD of % of predicted	67 + 25.2	44 + 8.8	49 + 21.9

SD, standard deviation; HRCT, high-resolution chest computer tomography.

## Data Availability

All data analyzed in this study are included in this published article.

## Appendix A

**Table A1:** Complete list of genes and PCD genes transcripts analyzed in our cohort.

Genes	Transcript Reference ^
*AK7*	NM_152327.3
*ARMC4 **	NM_018076.2
*C11orf70*	NM_032930.2
*CCDC103*	NM_213607.2
*CCDC114*	NM_144577.3
*CCDC151*	NM_145045.4
*CCDC39*	NM_181426.1
*CCDC40*	NM_017950.3
*CCDC65*	NM_033124.4
*CCNO*	NM_021147.4
*CEP164*	NM_014956.4
*CFAP298*	NM_021254.2
*DNAAF1*	NM_178452.4
*DNAAF2*	NM_018139.2
*DNAAF3*	NM_001256714.1
*DNAAF4*	NM_130810.3
*DNAAF5*	NM_017802.3
*DNAH1*	NM_015512.4
*DNAH11*	NM_001277115.1
*DNAH5*	NM_001369.2
*DNAH8*	NM_001206927.1
*DNAH9*	NM_001372.3
*DNAI1*	NM_012144.3; NM_001281428.1
*DNAI2*	NM_023036.4
*DNAJB13*	NM_153614.3
*DNAL1*	NM_031427.3
*DRC1*	NM_145038.3
*GAS8*	NM_001481.2
*LRRC56*	NM_198075.3
*LRRC6*	NM_012472.4
*MCIDAS*	NM_001190787.1
*NOTCH2 **	NM_024408.3
*OFD1*	NM_003611.2
*PIH1D3*	NM_001169154.1
*RPGR **	NM_000328.2
*RSPH1*	NM_080860.3
*RSPH3*	NM_031924.4
*RSPH4A*	NM_001010892.2
*RSPH9*	NM_152732.4
*SPAG1*	NM_172218.2
*ZMYND10*	NM_015896.2

^ Testing conducted by Invitae Corporation. * Gene limitations. *NOTCH2*: deletion/duplication and sequencing analysis is not offered for exons 1–4. *ARMC4*: Deletions/duplication and sequencing analysis was not conducted on exon nine. *RPGR*: Only the transcript associated with X-linked PCD was analyzed. *CFTR* testing was conducted: Sequencing analysis for exon seven includes only the 288 coding sequences of the gene + 10 base pairs.

**Table A2:** Complete list of VUS identified.

PCD Genes	Unique Variants
*DNAAF2*	c.58G > C (p.Val20Leu)
*DNAAF4*	c.186C > G (p.Ser62Arg)
*DNAH11*	c.8888C > A (p.Ser2963Tyr)
*DNAAF5*	c.969C > A (p.Asp323Glu)
*DNAAF3*	c.1672G > T (p.Glu558*)
*SPAG1*	c.2717A > G (p.Asp906Gly)
*CCDC103*	c.82C > T (p.Arg28Trp)
*CCDC40*	c.1109G > A (p.Arg370His)
*DNAI2*	c.1153G > A (p.Asp385Asn)
*CCNO*	c.685G > A (p.Gly229Ser)
*RSPH4A*	c.902A > C (p.Gln301Pro)
*CFAP298*	c.803C > T (p.Pro268Leu)
*RSPH1*	c.824G > A (p.Arg275Gln)
*RSPH1*	c.673C > T (p.Pro225Ser)
*CCDC103*	c.146G > C (p.Gly49Ala)
*DNAH5*	c.6409C > A (p.Leu2137Ile)
*DNAH5*	c.5413C > T (p.Arg1805Cys)
*DNAH5*	c.2577+5T > G (Intronic)
*DNAH5*	c.477C > T (Silent)
*DNAH5*	c.8398G > A (p.Val2800Ile)
*DNAAF5*	c.1471-3C > T (Intronic)
*DNAH11*	c.6273 + 12C > G (Intronic)
*DNAH11*	c.10379C > T (p.Thr3460Met)
*DNAH11*	c.11098G > C (p.Glu3700Gln)
*DNAH11*	c.3739A > C (p.Ile1247Leu)
*DNAH11*	c.5434G > A (p.Val1812Met)
*DNAH11*	c.3288G > A (p.Met1096Ile)
*DNAH11*	c.6154C > T (p.Leu2052Phe)
*DNAH11*	c.11678C > T (p.Thr3893Met)
*DNAH11*	c.13243A > C (p.Lys4415Gln)
*DNAH9*	c.3128A > T (p.His1043Leu)
*DNAH9*	c.3593G > C (p.Trp1198Ser)
*DNAH9*	c.4070C > A (p.Thr1357Lys)
*DNAH9*	c.1562A > T (p.Asp521Val)
*DNAH9*	c.1603G > A (p.Asp535Asn)
*DNAAF2*	c.10G > A (p.Ala4Thr)
*DNAAF2*	c.700C > T (p.Pro234Ser)
*DNAAF2*	c.1082_1087dup (p.Val361_Ala362dup)
*DNAH1*	c.7064C > T (p.Pro2355Leu)
*DNAH1*	c.3877G > A (p.Asp1293Asn)
*DNAH1*	c.1610G > A (p.Ser537Asn)
*DNAH1*	c.6650T > C (p.Leu2217Pro)
*DNAH1*	c.10208G > T (p.Gly3403Val)
*DNAH1*	c.7905G > C (p.Gln2635His)
*DNAH1*	c.9646C > G (p.Leu3216Val)
*DNAH1*	c.8491C > A (p.Arg2831Ser)
*DNAAF5*	c.412T > G (p.Cys138Gly)
*DNAAF5*	c.221C > A (p.Ala74Glu)
*DNAAF5*	c.1195G > A (p.Glu399Lys)
*DNAAF5*	c.454G > A (p.Ala152Thr)
*DNAH5*	c.12709G > T (p.Val4237Phe)
*DNAH5*	c.10731C > G (p.Asn3577Lys)
*DNAH5*	c.1438C > T (p.Leu480Phe)
*DNAH5*	c.12523G > A (p.Val4175Met)
*CCDC103*	c.355C > T (p.Arg119Cys)
*CCDC103*	c.197G > A (p.Gly66Glu)
*CCDC103*	c.301A > G (p.Thr101Ala)
*RSPH1*	c.835C > T (p.Arg279Trp)
*RSPH1*	c.635C > T (p.Thr212Ile)
*DNAH8*	c.8801T > C (p.Val2934Ala)
*DNAH8*	c.644G > A (p.Gly215Glu)
*DNAH8*	c.7114A > G (p.Met2372Val)
*DNAH8*	c.13607T > C (p.Leu4536Pro)
*DNAH8*	c.2096G > A (p.Arg699His)
*DNAH8*	c.9839A > T (p.Gln3280Leu)
*DNAH8*	c.9811G > T (p.Ala3271Ser)
*CFAP298*	c.534+4C > T (Intronic)
*DNAAF3*	c.623T > C (p.Leu208Pro)
*DNAAF3*	c.1615G > A (p.Gly539Arg)
*CCNO*	c.16C > A (p.Pro6Thr)
*CCDC40*	c.1076G > A (p.Arg359His)
*DNAI2*	c.487A > G (p.Arg163Gly)
*CCDC39*	c.1229A > G (p.Gln410Arg)
*CCDC39*	c.692T > C (p.Ile231Thr)
*CCDC39*	c.2057A > G (p.Asn686Ser)
*CCDC151*	c.619C > T (p.Arg207Trp)
*CCDC65*	c.1067T > C (p.Phe356Ser)
*CCDC65*	c.771G > T (p.Glu257Asp)
*RSPH3*	c.895G > C (p.Ala299Pro)
*NME8*	c.88A > G (p.Thr30Ala)
*GAS8*	c.1255G > A (p.Asp419Asn)
*LRRC56*	c.52G > T (p.Val18Phe)
*OFD1*	c.2584T > G (p.Ser862Ala)
*RSPH9*	c.98T > C (p.Met33Thr)
